# The Antiaggregative and Antiamyloidogenic Properties of Nanoparticles: A Promising Tool for the Treatment and Diagnostics of Neurodegenerative Diseases

**DOI:** 10.1155/2020/3534570

**Published:** 2020-10-13

**Authors:** Monika Pichla, Grzegorz Bartosz, Izabela Sadowska-Bartosz

**Affiliations:** ^1^Department of Analytical Biochemistry, Institute of Food Technology and Nutrition, College of Natural Sciences, Rzeszow University, Zelwerowicza Street 4, 35-601 Rzeszów, Poland; ^2^Department of Bioenergetics, Food Analysis and Microbiology, College of Natural Sciences, Rzeszow University, Zelwerowicza Street 4, 35-601 Rzeszów, Poland

## Abstract

Due to the progressive aging of the society, the prevalence and socioeconomic burden of neurodegenerative diseases are predicted to rise. The most common neurodegenerative disorders nowadays, such as Parkinson's disease, Alzheimer's disease, and amyotrophic lateral sclerosis, can be classified as proteinopathies. They can be either synucleinopathies, amyloidopathies, tauopathies, or TDP-43-related proteinopathies; thus, nanoparticles with a potential ability to inhibit pathological protein aggregation and/or degrade already existing aggregates can be a promising approach in the treatment of neurodegenerative diseases. As it turns out, nanoparticles can be a double-edged sword; they can either promote or inhibit protein aggregation, depending on coating, shape, size, surface charge, and concentration. In this review, we aim to emphasize the need of a breakthrough in the treatment of neurodegenerative disorders and draw attention to nanomaterials, as they can also serve as a diagnostic tool for protein aggregates or can be used in a high-throughput screening for novel antiaggregative compounds.

## 1. Introduction

Undoubtedly, the progress in medical and biological studies has led to increased quality of life and extension of life span. Furthermore, the overall fertility has dropped and these two factors contribute to the aging of the society. Due to this phenomenon, the increase in prevalence of neurodegenerative diseases is predicted to be more visible in the future than it currently is. According to the World Health Organization, it is projected that the number of people aged ≥ 65 will grow from about 524 million in 2010 to around 1.5 billion in 2050 [1]. Neurodegenerative diseases impose burden not only on people affected by this disorder but also on their caregivers. There are three major neurodegenerative diseases whose pervasiveness and incidence significantly rise with age.

First of them and the most common one is Alzheimer's disease (AD), which affects approximately 30% of people aged 85 or older. After the age of 85, the incidence of AD rises gradually from 6 to 8% per year, in contrast to the 0.5% rise per year when peoples' age ranges between 65 and 75 [2]. The second most common is Parkinson's disease (PD), which affects 10-15 per 100 000 people annually [3]. Its prevalence has been more than two times higher in 2016 (6.1 million cases) comparing to 2010 (2.5 million cases) and may reach 2% among people aged ≥ 65. Consequently, it is estimated that in 2050, there will be more than 12 million cases of PD worldwide [4]. Subsequently, amyotrophic lateral sclerosis's (ALS) annual incidence is approximately 1-2.6 new cases per 100 000 persons. This disease is characterized by rapid progression with average survival 3-4 years from onset, whereas the average age of onset nowadays is 59-60 years [5].

Indeed, there is a variety of symptoms of the aforementioned neurodegenerative diseases, but the exact pathophysiology of these conditions is still elusive. Nevertheless, it should be emphasized that they have some common pathogenic features. Among them, genetic [6] and environmental [7] factors can be listed. Yet, the most classical feature of all these diseases is protein misfolding in specific brain regions; thus, these disorders can be classified as proteinopathies (Table 1). The hallmark of proteinopathies is either intra- or extracellular accumulation of aggregates in the central nervous system that are abundant in *β*-sheets. In these diseases, altered forms of proteins, which play a physiological role, accumulate in the brain. They turn out to have pathological functions after modifications of their 3D structure, which in consequence leads to self-aggregation, aggregate growth, and eventually precipitation [8].

Unfortunately, the current and only available treatment of neurodegenerative diseases is strictly symptomatic. Treatment of PD has not significantly changed over decades: *L-*DOPA treatment is a gold standard for 60 years so far. Apart from levodopa-carbidopa preparations, other dopamine agonists, monoamine oxidase-B inhibitors, cholinesterase inhibitors, and selective serotonin and norepinephrine reuptake inhibitors are also used as a drug regimen [13, 14]. The treatment of AD is not much more sophisticated and is based on cholinesterase inhibitors and NDMA receptor agonist, namely, memantine. It addresses not only the behavioural and cognitive symptoms but also covers for functional ones [15]. A review of treatments for AD in clinical trials can be found in a recent article [16], demonstrating that there is no effective antiaggregative treatment so far. Similarly, the information about PD drugs in clinical trials can be found in another review [17]. When it comes to ALS, there is only one FDA approved drug—riluzole, which has a glutamine agonist activity and extends the survival of patients by only 2-3 months [18, 19].

**Figure 1:**
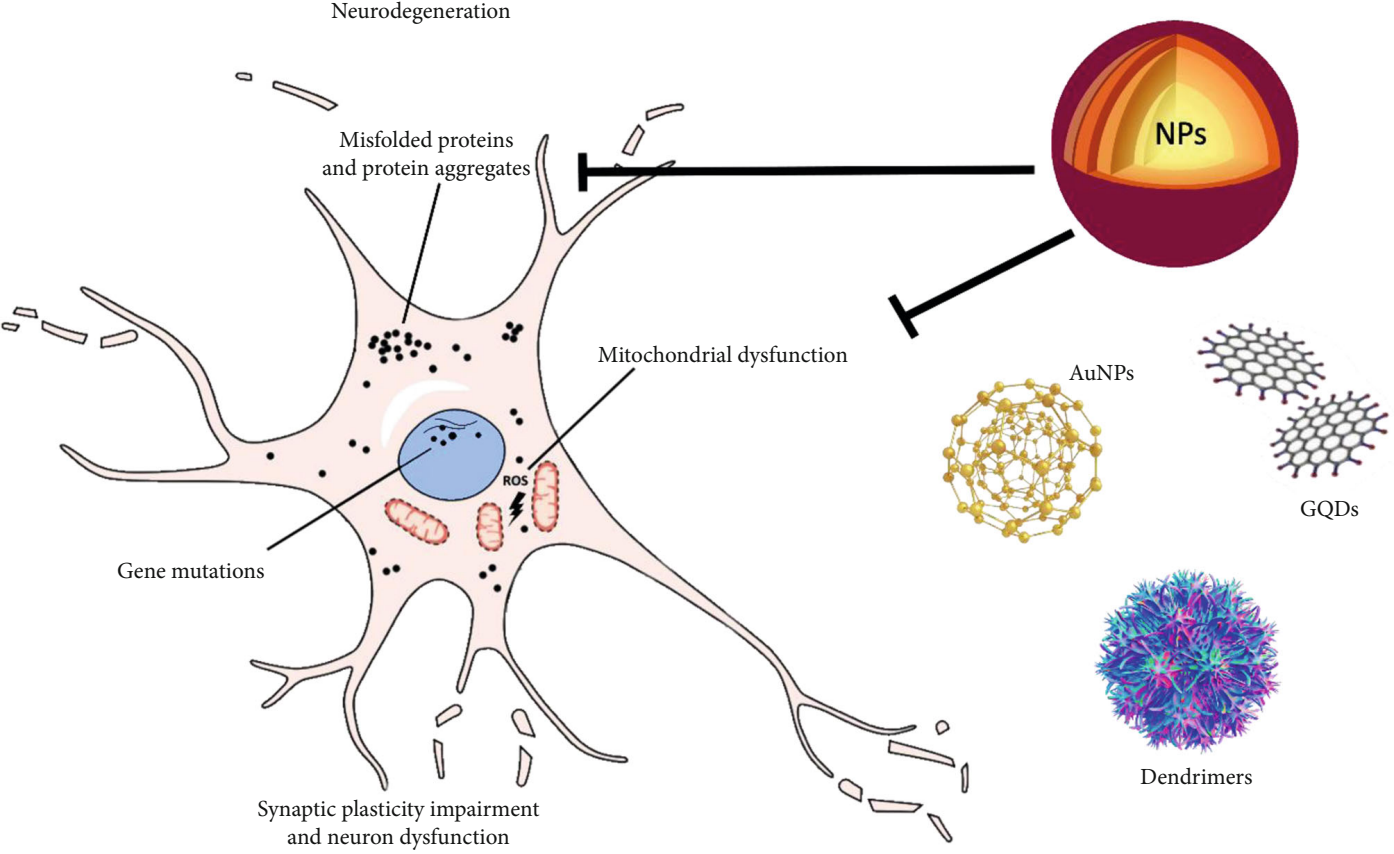
Impact of NPs on neurodegeneration hallmarks.

Due to the abovementioned facts, in this review, we aim to highlight the burden of neurodegenerative diseases and discuss novel approaches to their treatment using nanomaterials (Figure 1). Furthermore, we would like to point out the versatility and impact of the nanoparticles used to combat proteinopathies, on pathological protein aggregation.

## 2. Protein Aggregation in Neurodegeneration

A body of evidence suggests that the accumulation and transmission of *α*-synuclein (*α*-syn) aggregates in the midbrain are highly associated with the pathogenesis of PD [20]. *α*-Synuclein is a presynaptic protein, which probably plays a regulatory function in modulation of synaptic plasticity, control of presynaptic vesicle pool size, release of neurotransmitters, and vesicle recycling. Its structure can be divided into three regions: an amphiphilic N-terminus, an acidic C-terminus, and a hydrophobic central domain, which is known as the nonamyloid *β* component (NAC). The NAC region is crucial for *α*-syn aggregation and formation of *β*-sheet fibrils, which are the main elements of Lewy bodies [21]. Studies showed that electrostatic forces play a crucial role in *α*-syn fibrillation; thus, this process can be obstructed by charged nanoparticles [22].

Yet, interestingly, the exact molecular mechanism, time of occurrence, and influence of protein misfolding on the onset and/or progression of these particular diseases are still beyond reach. According to Janezic et al. who introduced a new mouse model for PD studies, neurophysiological changes forerun and are not driven by *α*-syn aggregate formation [23]. Nevertheless, the search for antiaggregative agents is still highly desirable.

Amyloid *β* peptide has a leading role in the onset and progression of AD. In this disease, amyloid plaques containing aggregated amyloid-*β* protein (A*β*) are surrounded by morphologically altered neurons, cause synapse and memory loss, and induce neurotoxicity [24].

As a matter of fact, A*β* is physiologically present and derives from the amyloid precursor protein (APP), which is implicated in regulation of synapse formation. Unfortunately, under particular circumstances, it starts to aggregate and initiates the disease progression [25]. Amyloid-*β* protein monomers tend to aggregate into several forms, namely, soluble oligomers, protofibrils, and insoluble amyloid fibrils which can further aggregate into amyloid plaques. This process is accompanied by oxidative stress, leading to the formation of oxidized proteins and lipid peroxidation. Products of lipid peroxidation, especially 4-hydroxynonenal, can in turn disrupt function of glucose and glutamate transporters and of ion-dependent ATPases [26]. Therefore, A*β* advocates synaptic membrane depolarization, uncontrolled Ca^2+^ influx, and mitochondrial damage, which cause undesirable changes in cellular activity [27].

Additionally, tau protein is being hyperphosphorylated because of changes in protein kinase activity, which are a result of A*β* aggregation. The hyperphosphorylated form of tau protein becomes a core of neurofibrillary tangle (NFT) formation, whereas physiologically tau protein fosters the assemblance of tubulin into microtubules and helps to maintain their stability. The link between the existence of NFTs and neuronal dysfunction is straightforward. Notwithstanding, the relation of A*β* and NTFs is intertwined, because the inhibition of tau generation can impact production of A*β* and its derivatives [28].

## 3. Nanoparticles as Therapeutics of the Future

**Figure 2:**
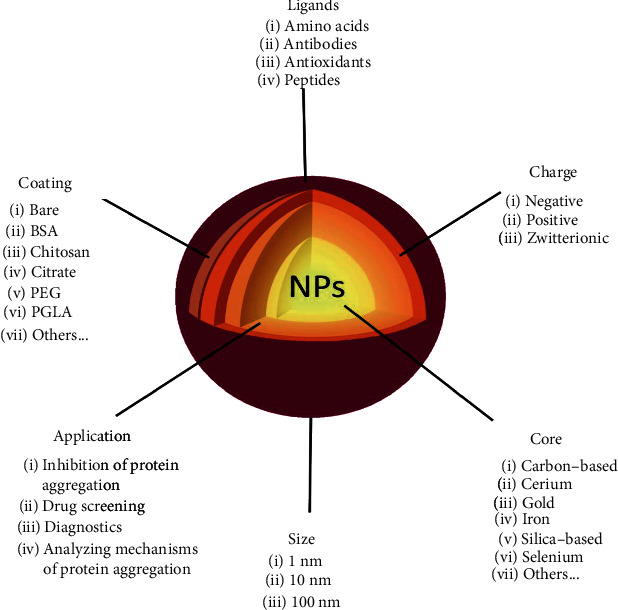
Possible modifications of NPs for prevention and diagnostics of neurodegeneration.

A nanoparticle (NP) is defined as a particle of matter that is between 1 and 100 nanometres in at least one dimension. Nanoparticles arose as attractive tools for both therapeutic and diagnostic applications, especially in imaging, diagnostics, and drug delivery. They can be synthesized from a broad range of materials, such as polymers, metals, or carbon-based molecules. NPs are also highly functional because of the ease with which their shape, size, and surface properties can be modified. Furthermore, NP properties can be also altered by attachment of other substances to the surface or their entrapment within the NP cavities, if these exist (Figure 2) [29].

### 3.1. Graphene Quantum Dots

**Figure 3:**
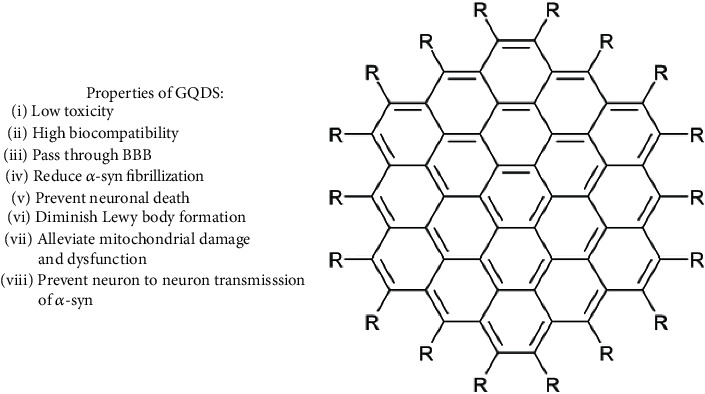
The structure and properties of GQDs.

Graphene quantum dots (GQDs) are less than 100 nm in size and are made of single- or few-layer graphene (Figure 3). They have been widely used in nanobiomedicine by virtue of their low cytotoxicity and high biocompatibility [30]. The group of Kim et al. demonstrated that GQDs were able to pass through the BBB. In the brain, they reduced *α*-syn fibrillization and triggered fibril disaggregation in a time-dependent manner by direct interaction with mature fibrils. The binding between GQDs and *α*-syn is driven by negatively charged carboxyl groups of GDQs and the positively charged *α*-syn region. Furthermore, these GQDs did not manifest any long-term toxicity *in vivo* and *in vitro* and also were able to prevent neuronal death, diminish Lewy body and Lewy neurite formation, and alleviate mitochondrial damage and dysfunction, and last but not least, they have the ability to prevent neuron to neuron transmission of pathological *α*-syn. Moreover, experiments performed on a mouse model showed that GQD protected against *α*-syn preformed fibril-induced loss of dopaminergic neurons and alleviated motor deficits [31].

With regard to AD, GQDs were also used to inhibit A*β* aggregation. The *β*-amyloid peptide consists of 39-42 amino acids, where several regions can be defined. The His13-Lys16 (HHQK) region plays a significant role in oligomerization and fibril formation. This region is a crucial component of glycosaminoglycan (GAG) binding site, which facilitates a conformational change of A*β* from soluble and unordered *α*-helix to stable *β*-sheet [32]. A construct composed of GQDs and tramiprosate, a mimic of GAGs, which specifically binds to HQQK motif and inhibits A*β* peptide aggregation, showed an inhibition of A*β* aggregation driven by breaking *β*-sheets. Furthermore, GQDs combined with tramiprosate evidently protected PC12 cells from A*β*-induced cytotoxicity, meanwhile exhibiting a synergistic effect [33].

### 3.2. Dendrimers

**Figure 4:**
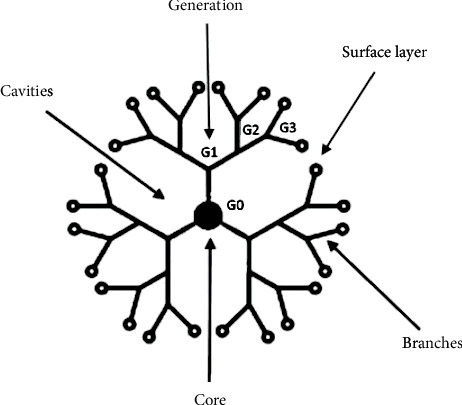
The structure of dendrimers.

Dendrimers are highly branched, tree-like polymers with unique properties thanks to their terminal functional surface groups (Figure 4). The size, shape, and surface charge change with an increase in generation. Dendrimers are highly functional because of simplicity of modifying their biological and/or physicochemical properties [34, 35]. There is evidence that generations 3, 4, and 5 of PAMAM dendrimers are able to interfere with A*β* aggregation by blocking growth of new fibrils and breaking the existing ones in a concentration- and generation-dependent manner: the higher the dendrimer concentration and generation, the lower number of new fibrils [36]. A similar impact of dendrimers on *α*-syn aggregates has been observed. The dendrimers inhibit formation of *β*-sheet structures and disrupt remaining *β*-sheets or the agglomerates, in concentration and generation axis [37]. Furthermore, only full-generation PAMAM dendrimers, which have the cationic amino groups on their surface, were able to interact with the basic amino acid N-terminal region of *α*-syn responsible for the *β*-sheet formation and protein aggregation, contrary to half-generation PAMAM dendrimers [38]. Third and fifth generations of polylysine dendrimers obstructed amyloid aggregation in solution, whereas generation 3 dendrimers also protected SH-SY5Y cells against amyloid-induced toxicity [39].

### 3.3. Metal Nanoparticles

Cerium oxide nanoparticles (CeO_2_ NPs) or nanoceria are multifaceted polymers. They are characterized by good bioavailability and ability to mimic superoxide dismutase or catalase activity. They are quite potent ROS and nitric oxide scavengers. The antioxidant properties of CeO_2_ NPs are linked to the Ce^3+^/Ce^4+^ redox shift. Additionally, research shows that nanoceria are able to protect neurons against A*β*-induced mitochondrial fragmentation and also reduce DRP-1 hyperphosphorylation on Ser616, which is related with AD and neurodegeneration. Inhibiting this posttranslational modification turns out to be a potential mechanism of mitochondrial preservation [40]. Beyond that, another group of researchers studied the influence of CeO_2_ NPs in a yeast model of PD. Nanoceria significantly increased the viability of yeast cells expressing *α*-syn. In addition, these NPs decreased *α*-syn-induced ROS production and alleviated mitochondrial dysfunction and fragmentation. The most probable mechanism of inhibiting the formation of *α*-syn aggregates occurs by a direct interaction of these nanoceria with *α*-syn monomers or oligomers; hence, their miscellaneous properties are also exhibited by the ability to adsorb *α*-syn on the nanoparticle surface [41].

Gold nanoparticles (AuNPs) have been extensively used in biomedicine because of their great biocompatibility, chemical inertness, and effortless size control. AuNPs are also able to abrogate aggregation of pathological proteins. Nevertheless, they may be toxic; toxicity of gold NPs significantly depends on their size, charge, and coating. Large AuNPs (36 nm and 18 nm) increase A*β* fibrillation, whereas small ones are able to delay (6 nm) or utterly inhibit (1.9 nm) this process [42]. Particularly, smaller, anionic NPs exhibit better ability to halt protein aggregation. The researchers have studied four different coatings (citrate, poly(acrylic acid) (PAA), poly(allylamine) hydrochloride (PAH), or polyelectrolyte surfaces) and three different sizes of AuNPs (8 nm, 18 nm, and 40 nm). The results altogether demonstrated that PAA-coated, 18 nm AuNPs exhibited superiority in the inhibition of A*β* aggregation and were the least toxic towards human neuroblastoma SH-SY5Y cells [43]. In order to improve the ability of AuNPs to cross the BBB, Prades et al. created an AuNP conjugated with two peptides, where one of the peptide sequences was designed to interact with the transferrin receptor. The authors suggest that this platform can increase the efficiency of drug delivery into the brain [44]. Noteworthily, natural compounds are also able to obstruct amyloid fibrillation and break existing amyloid fibrils, one of which is curcumin [45]. Because of its hydrophobicity and thus insolubility in water, curcumin has to be conjugated with other compounds [46]. Water-soluble curcumin-functionalized gold nanoparticles turned out to efficiently inhibit amyloid fibrillation, but also to break and dissolve A*β* fibrils. Furthermore, these curcumin-AuNPs protect neuro2a cells from A*β*_1-40_ fibril-induced cytotoxicity, giving nearly doubled improvement in viability. It is suspected that the great inhibitory efficiency is a result of nanoparticle binding to the fibrils *via* curcumin moiety and disrupting the elongation phase of fibrillation [47].

### 3.4. Antioxidant-Loaded NPs

**Figure 5:**
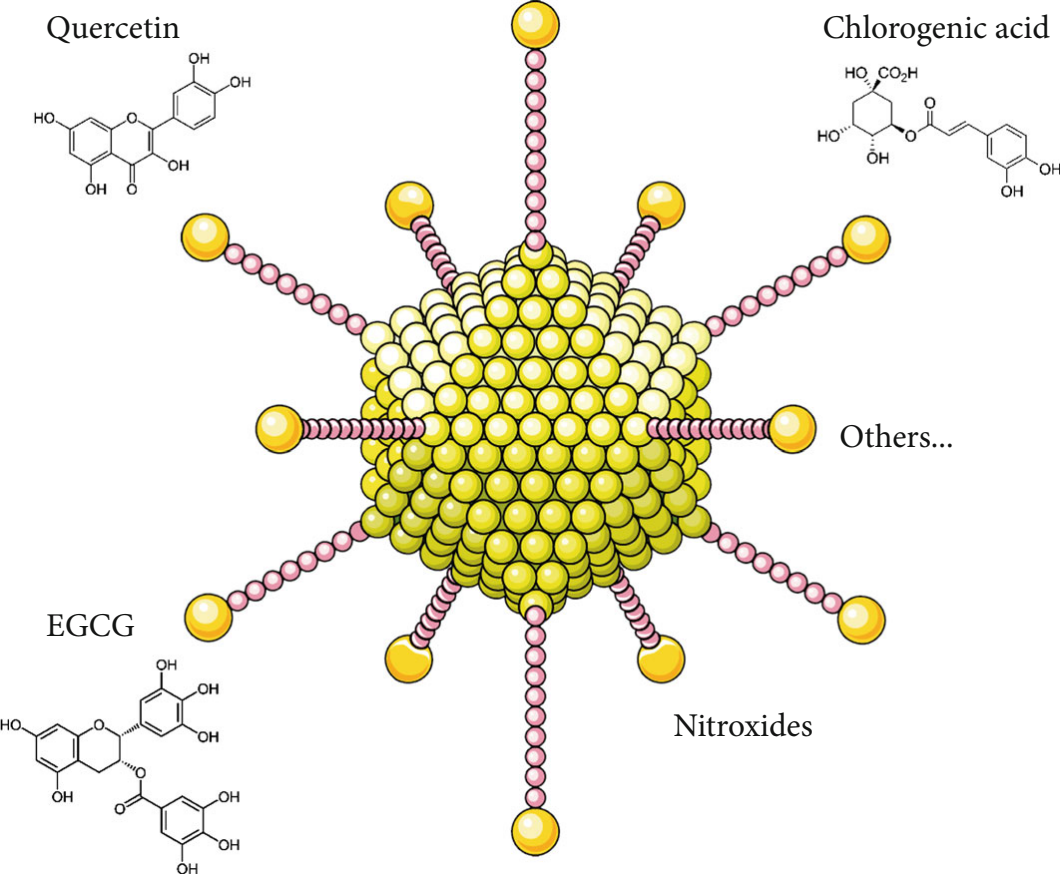
Possible ligands of antioxidant-loaded nanoparticles.

Apart from the abovementioned example, other phytochemicals have also arisen as useful in prohibiting pathological protein aggregation regarding neurodegenerative diseases (Figure 5). Among them, baicalein [48], chlorogenic acid [49], gallic acid [50], and many other natural compounds [51] are able to inhibit the formation of *α*-syn aggregates and/or even disaggregate existing ones. Selenium nanoparticles (SeNPs) turned out to be an effective carrier of antioxidants. Their peculiar biomedical applications and wide range of therapeutical properties are ascribed mainly to the ability to modulate redox state. Moreover, SeNPs show low toxicity and great biodegradability *in vivo* [52]. Yang et al. investigated anti-A*β*-aggregative and antioxidative properties of SeNPs conjugated with chlorogenic acid (CGASeNPs). These authors hypothesized that binding CGA with nanoparticles will improve its bioavailability and stability. They proved that antiaggregative properties of CGASeNPs are contributed by their ability to bind A*β*_40_ on their surface. Furthermore, CGASeNPs effectively scavenged ROS and protected PC12 cells against A*β*-induced toxicity [53]. Likewise, the same group designed SeNPs modified with resveratrol and tested their properties against ion metal-induced A*β*42 aggregation. They obtained similar effects as described above, i.e., that resveratrol and SeNPs exhibit synergistic effect regarding the inhibition of pathological protein aggregation [54]. A nanocomposite engineered from quercetin, SeNPs, and polysorbate 80 can serve as another example of SeNPs combined with antioxidants. *In vitro* analyses showed that the nanocomposite exhibited greater solubility in water comparing to quercetin *per se*, which has poor aqueous solubility. On top of that, such nanocomposite had an exceptional antioxidative activity, inhibited A*β*_1-42_ monomer aggregation, and protected PC12 cells from hydrogen peroxide-induced cell death [55]. Zhang et al. studied both EGCG-SeNPs and NPs conjugated with EGCG and Tet-1 peptide. Tet-1-EGCG-SeNPs showed better efficacy comparing to NPs without the peptide. Both types of NPs not only protected PC-12 cells against amyloid-induced cytotoxicity and inhibited A*β* fibrillation but were also able to dissociate existing fibrils into nontoxic monomeric state. Nevertheless, peptide-containing NPs had overall better performance due to increased neuronal targeting efficiency *in vitro* [56]. NPs loaded with other antioxidants, namely, ferulic acid (as a powerful anti-inflammatory agent) and tannic acid (acting as an inhibitor of *α*-syn fibrillation), exhibited potent inhibitory effect on *α*-syn aggregation, diminished proinflammatory responses, and reduced oxidative stress caused by *α*-syn [57]. Additionally, curcumin-loaded NPs inhibited amyloid-like aggregation of superoxide dismutase (SOD) 1, which occurs in about 20% of familial ALS cases [58].

Nanoparticles loaded with synthetic antioxidants can also serve as antiaggregative agents. Nitroxides exhibited better efficacy in prevention of nitration reactions and were more reactive than natural antioxidant, vitamin E [59]. It has been established that nitroxide-containing redox NPs are able to alleviate typical aspects of neurodegenerative diseases, namely, protect cells against oxidative stress, improve mitochondrial function, and inhibit A*β* aggregation [60, 61].

## 4. Other Therapeutic Approaches

Unquestionably, transition metals are among the main culprits of pathological protein accumulation. Moreover, they widely contribute to an altered redox state; thus, chelators might bring alleviation of the toxic activity of these metals. Liu et al. created a chelating nanoparticle, in a nutshell—a NP conjugated with 2-methyl-N-(2′-aminoethyl)-3-hydroxyl-4-pyridinone. This construct significantly inhibited A*β* aggregation, protected human cortical neuronal cells from A*β*-induced cytotoxicity, and had no impact on cell proliferation [62].

Given the fact that the nanoparticle efficacy in inhibiting protein aggregation greatly depends on the surface charge, the use of amino acids as coating agents is not surprising; they may enhance biocompatibility of nanoparticles. It is mainly due to the fact that amino acids are zwitterionic. Antosova et al. proved that amino acid-coated superparamagnetic nanoparticles can be quite a powerful tool for treatment of amyloidopathies. The group showed that tryptophan-coated NPs exhibited the best antiaggregative properties [63]. Furthermore, others demonstrated that histidine-coated nanoparticles can completely suppress amyloid fibril formation [64]. Moreover, lysine-coated Fe_3_O_4_ NPs were less toxic than bare iron oxide NPs, strongly bound to monomeric *α*-syn, and inhibited the early phases of its aggregation [65].

NPs can be also used as safer carriers for gene therapy, instead of viral vectors. Niu et al. created multifunctional magnetic nanoparticles which are a complex platform that combines elements of cell targeting, controlled drug release, and gene therapy. The authors developed a NP that interferes with *α*-syn synthesis by shRNA, hence alleviating its toxic effect, so cell death is inhibited both *in vitro* and *in vivo* [66].

## 5. The Dark Side of the Nanoparticles with a Useful Outcome

Despite undoubted success of some nanoparticles as promising antineurodegenerative compounds, it is important to mention that there is also data on their possible contribution to the disease progression. A plethora of evidence suggests that the nanostructures can influence protein fibrillation depending on various conditions, including the coating, size, surface charge, and concentration. Such discrepancy has been seen for example in silica-based nanoparticles, where positively charged silica nanoparticles inhibited *α*-syn fibrillation and negatively charged one had an opposite effect [67]. Also, it was also established that SiO_2_NPs upregulate *α*-syn expression, inhibit protein levels of the ubiquitin-proteasome system, and induce autophagy by interference in the PI3K-Akt-mTOR signalling pathway [68].

Contrary to that, negatively charged gold nanoparticles act as chaperones and prevent A*β* fibrillation [69]. Yet, regarding *α*-syn, the opposite effect was seen: gold nanoparticles are also a double-edged sword. Citrate-capped (negatively charged) AuNPs speeded up the formation of *α*-syn aggregates in nanomolar concentrations, and time of the nucleation phase was dependent on the surface availability. The smaller the NPs (10-14 nm), the more aggregate growth acceleration, whereas particular sizes (22 nm) were able to inhibit the fibrils' growth; thus, in summary, the AuNP aggregative properties hinge on their size and concentration [70].

Nowadays, numerous NPs have been used in a variety of fields, namely, electronics, pharmaceuticals, cosmetics, and fabrics; hence, their toxicity has started to be more widely observed and studies on the health risks are a bit behind the prompt development of nanotechnology, regardless of a body of evidence of toxic effects of NPs, both *in vitro* and *in vivo* [71]. For example, Shah et al. prove that nanoscale-alumina can accumulate in the brains of exposed animals and thus induce oxidative stress and neurodegeneration. It promoted the production of toxic A*β* through the amyloidogenic pathway, caused overexpression of APP, and increased the *β*-secretase BACE1 activity that boosted the formation of A*β* aggregates. Their findings suggest that exposure to nanoalumina might increase the probability of the neurodegenerative disease onset [72].

It is worth mentioning that TiO_2_NPs are commonly used in numerous daily use products like cosmetics or antiseptic agents. These NPs turned up to induce *α*-syn fibrillation *via* shortening its nucleation process and may contribute to PD onset [73]. Additionally, there is a positive correlation between *α*-syn expression levels and TiO_2_NP concentration [74]. Moreover, exposure of wild-type mice to inhalation of nickel-containing NP air for 3 h increased both A*β*40 and A*β*42 amyloid peptide levels in the brain by 72-129% [75].

Moreover, Yarjanli et al. gave excellent examples of the role of iron in neurodegeneration. They speculated whether iron ions released from the NPs are capable to activate positive feedback loop among iron accumulation. First and foremost, there is evidence that released iron ions can support Fenton's reaction and produce ROS from hydrogen peroxide and superoxide. Further than that, iron NPs can decrease GSH content, which may lead to increased oxidative stress and mitochondrial degradation. Due to these factors, it is not a surprise that these NPs can boost protein aggregation. Nevertheless, the authors emphasize that the toxicity of iron NPs is dependent on their size, shape, surface charge, coating, functional groups, and concentration and their utility must be considered in regard to these aforementioned aspects [76].

In any case, the knowledge about the diverse nature of nanoparticles was used to look for their other applications in the field of neurodegenerative disorders. For the case in point, nanoparticle-induced protein fibrillation can be employed as a fast screening method for novel potential antiaggregative compounds [77] and also as a methodology for rapid detection of protein aggregation that can be used to analyze the fibrillation process as well [78]. Likewise, NPs can serve as advanced, real-time screening platform which will help to identify various mechanisms of A*β* aggregation [79].

Here, SOD1-functionalized AuNPs served as a colorimetric detection platform for SOD1 aggregate evaluation. The test is simple and sensitive comparing to other methods as it is based on absorbance; thus, such a sensor system can serve as a diagnostic tool of SOD1 aggregates which are a hallmark of a fraction of familial ALS [80].

A step further, some nanoparticles might be designed not only to inhibit aggregation of pathological proteins but also to serve as a diagnostic tool. The results presented by Skaat et al. indicate that the conjugation of a BAM10 antibody to the near-infrared fluorescent Fe_3_O_4_ nanoparticles not only significantly hinders A*β*40 fibrillation but also acts as their marker; thus, the aggregates can be detected by MRI or fluorescence imaging [81]. Another example of “traceable” and successful antineurodegenerative NPs is the superparamagnetic iron oxide nanoparticles conjugated with two cell targeting molecules—a peptide with strong affinity to transferrin receptor used in order to enable NP crossing the BBB and mazindol, a dopamine inhibitor which stimulates dopamine transporter internalization to facilitate specific internalization to dopaminergic neurons. EGCG attached to this NP prevents *α*-syn aggregation [82].

## 6. Conclusions

This review gives an insight into the burden and predictions of the prevalence of the most common neurodegenerative diseases and the lack of effective treatment. Contemporary regimen is solely symptomatic; thus, we wanted to point out the emerging significance of nanoparticles as a promising approach in the treatment and diagnostics of these disorders. Despite the complexity of mechanisms underlying neurodegenerative diseases, some pathological aspects tend to overlap; thus, nanoparticles can act on many levels. Further, both *in vitro* and *in vivo* studies are extremely important to the discovery of the most efficient treatment of these diseases.

## Figures and Tables

**Table 1:** Characteristics of the most common neurodegenerative diseases.

Disease	Hallmarks	Genetic factors	Ref.
Alzheimer's disease	(i) Senile plaques comprising deposits of *β*-amyloid (ii) Intracellular neurofibrillary tangles (iii) Tau protein aggregation (iv) Neuronal loss	(i) Presence of specific allelic variants of *APOE* gene (*ε*2, *ε*3, and *ε*4) (ii) Apo*ε*4 allele specifically in sporadic form of AD (iii) Mutations of gene coding for amyloid precursor protein (*APP*), presenilin 1, and presenilin 2 (n or PSEN2) in the familial form of AD	[9]
Parkinson's disease	(i) Presence of Lewy bodies—neuronal inclusions of fibrillated aggregates comprising *α*-synuclein and ubiquitin (ii) Degeneration and loss of dopaminergic neurons especially in *substantia nigra pars compacta* (iii) Dopamine deficiency	(i) Gene mutations: *SNCA*, *Parkin*, *PINK1*, *LRRK2*, *DJ-1*, *VPS35*, *PLA2G6*, *DCTN1*, *FBX07*, and *ATP13A2*	[9]
Amyotrophic lateral sclerosis	(i) Presence of ubiquitinated inclusions comprising TDP-43, FUS, OPTN, ATXN2, C9ORF72, and UBQLN2 (ii) Slow and progressive degeneration and loss of motor neurons (iii) Neuroinflammation	(i) Gene mutations: *SOD1*, *C9ORF72*, *FUS*, and *TARDBP*	[9–12]

## Abbreviations

A*β*: Amyloid-*β* protein
AD: Alzheimer's disease
ALS: Amyotrophic lateral sclerosis
APP: Amyloid precursor protein
AuNPs: Gold nanoparticles
BBB: Blood-brain barrier
CGASeNPs: SeNPs conjugated with chlorogenic acid
*L*-DOPA: L-3,4-Dihydroxyphenylalanine
EGCG: Epigallocatechin gallate
GAGs: Glycosaminoglycans
GQDs: Graphene quantum dots
GSH: Glutathione
NAC: Nonamyloid *β* component
NFTs: Neurofibrillary tangles
NMDA: *N*-Methyl-D-aspartate
NPs: Nanoparticles
PAMAM: Poly(amidoamine)
PD: Parkinson's disease
ROS: Reactive oxygen species
SeNPs: Selenium nanoparticles
SOD: Superoxide dismutase
*α*-syn: Alpha-synuclein.
